# Increasing lung screening uptake: Exploring people who smoke and their family members’ concerns and recommendations regarding screening invitations

**DOI:** 10.18332/tpc/194630

**Published:** 2025-01-10

**Authors:** Rebecca Bell-Williams, Manpreet Bains, Rebecca Thorley, Emma O. Dowd, David Baldwin, Rachael Murray

**Affiliations:** 1 Faculty of Medicine and Health Sciences, University of Nottingham, Nottingham, United Kingdom

**Keywords:** lung cancer screening, uptake, eligibility, invitational materials, family members

## Abstract

**INTRODUCTION:**

Challenges with designing invitation materials and accessing high risk communities are all factors in encouraging attendance at lung screening. This study focused on ways to improve participation in those potentially eligible for lung screening.

**METHODS:**

A total of 50 qualitative interviews and 4 focus groups (n=17) were undertaken with people aged 50–75 years from East Midlands, UK. Individuals were purposively sampled to include those who were potential lung screening participants (PSP: n=39) and family members (FM: n=11) of people who smoke, and therefore potentially eligible for participation. Semi-structured discussion guides explored views on lung screening and ways to support uptake. Interviews and focus groups were audio-recorded, transcribed verbatim and analyzed using the framework approach.

**RESULTS:**

Data highlighted a number of novel findings. Themes centered on involving family members in encouraging lung screening attendance, appropriate phrasing around differing types of tobacco use and considering people who do not smoke within the context of lung screening eligibility.

**CONCLUSIONS:**

Exploring the use of family members in encouraging attendance in lung screening may be a valuable, but as yet underused approach. Additional phrasing around varying types of tobacco use may help to clarify invitational materials. Clearer eligibility guidelines regarding lung screening may clarify the role of smoking in lung screening.

## INTRODUCTION

Lung cancer is the leading cause of cancer deaths among men and women with an estimated 1.8 million new diagnoses worldwide and 1.6 million deaths annually^1^. In the UK, there are approximately 45525 new cases of lung cancer and over 35000 deaths each year^2^. Lung cancer survival is known to be poor in England compared with equally well-resourced healthcare systems^3,4^, because around 70% of patients first present to specialist care with advanced disease, and current treatment at this stage has very little effect on mortality. Earlier diagnosis is therefore essential, and the most promising approach is screening with low dose computed tomography (LDCT)^5^. In LDCT screening trials, over 70% of lung cancers are detected at an early stage (I or II)^5-8^. Despite the efficacy of lung cancer screening, there is evidence that the people most at risk of lung cancer are the least likely to attend screening, something that is expected from previous work in smoking cessation^9,10^. Exploring ways in which informed participation by those most at risk of lung cancer could be maximized is warranted, if programs are to show clinical and cost effectiveness.

Challenges with designing invitation materials and ensuring that high risk communities are accessing and understanding these resources are all factors in encouraging attendance^11,12^. In encouraging high-risk people to consider participating in screening programs, it is important that they are approached using the most effective methods; a tailored approach has the potential to do this. This study explores attitudes towards lung cancer screening with high-risk participants, and how new approaches could be designed to target, reach and engage at-risk individuals. The role of family in decision making regarding cancer care has been well documented^13,14^; however, this has primarily been in relation to those already engaged in treatment and there is little investigation into the role that family members can play in encouraging uptake of initial screening. Nevertheless, anecdotal discussion from cancer charities and research from some cancer studies, indicate that family members can play an important role in this stage^15^. To our knowledge little formal research in relation to lung cancer has been undertaken in this area despite the acknowledgement of its importance^16,17^ and as such this study provides some valuable initial data.

## METHODS

### Design

The study follows a qualitative approach and consists of interviews and focus groups. The study was approved by the University of Nottingham’s Faculty of Medicine and Health Sciences Ethics Committee (236-0421). The two phases involved interviews with individuals to identify positive and negative aspects of the invitation to lung screening process. This was followed by focus groups to discuss and review some potential screening materials in more detail.

### Recruitment and sampling

People who smoke, individuals with a smoking history, aged 50–75 years, and family members of people who smoke, were invited via adverts posted in the East Midlands, UK (Facebook adverts, local community centers, and snowball sampling), to take part in semi-structured telephone interviews or focus groups, on two occasions. Recruitment materials prompted interested individuals to contact the researcher to express interest. Individuals who wished to participate were provided with a detailed participant information sheet, had the opportunity to ask questions prior to arranging an interview and signing an informed consent form. Experian Mosaic Public Sector Groups (MPSG) data^18^ were also recorded. MPSG is a sociodemographic classification system that covers the UK; it collects data regarding lifestyles, consumer behavior and culture of neighborhoods categorized using postcode areas. MPSG data were used in this study to ensure representation of people from different groups at risk of developing lung cancer. MPSG data were also utilized to potentially help to indicate any societal segmentation relating to preferred routes of communication.

### Data collection and procedure

Over a six-month period, 50 qualitative one-to-one interviews lasting approximately one hour took place either online or over the phone. Prior to starting data collection, matters pertaining to the purpose of the study, consent, data management and withdrawal were reiterated, and individuals were given the opportunity to ask questions.

A semi-structured discussion guide was developed and covered the following topics: views on the type and content of screening invitations, knowledge of and barriers to lung screening, ways to encourage attendance at lung screening, and the role of family members in health-based decision making. Interview data were analyzed, and findings informed a second phase of data collection – four focus groups, three of which were online and one face-to-face which explored promotional, invitational and informational materials on lung screening further. In the focus groups, resources that had been developed based on participant feedback were discussed alongside invitational materials sourced from other screening programs.

### Analysis

All qualitative data collected were audio-recorded, transcribed verbatim and analyzed using the framework approach^19^. Following receipt of transcripts, data were checked for accuracy and personal identifiers were removed. NVivo 12.0 was used to facilitate data management and analysis. Data were coded both according to *a priori* themes (based on aims) and inductively. Initial readings facilitated familiarization and led to the generation of initial codes. Further reading and immersion led to the development of more substantive themes and subthemes, resulting in the generation of an analytical framework. Data were then indexed according to the identified thematic framework. A sub-sample of data was double coded (MB, RT, RM) to ensure validity of interpretations. Finally, themes were discussed and agreed upon between the research team, allowing clarification of the final framework that was then applied across all the transcripts. Data were then charted according to each theme to facilitate interpretation, synthesis, and reporting. These codes were then developed into themes using framework analysis. Once the key themes and subthemes were identified they were then used to inform the development of the new invitational material used in the focus group discussions. The data collected on these materials from focus group participants were also then processed as above and themes developed using the framework approach.

## RESULTS

### Participant characteristics

Fifty individuals were interviewed and consisted of 39 potential screening participants, 26 of which were over 55 years of age and current or previous smokers, and therefore currently eligible for screening. The remaining 11 interviews took place with family members of people who smoke, 4 of which were over 55 years and only 2 of which were previous smokers (the remaining 9 had never smoked). Focus group participants consisted of those who had been involved in the first round of data collection and had agreed to take part in either a face-to-face focus group or a virtual focus group. Four focus groups were undertaken (n=17) and consisted of ex-smokers over 50 years of age (n=10), current smokers over 50 years of age (n= 5) and family members who lived with people who smoke (n= 2). The focus group participants consisted of 12 females and 5 males, with 41% identifying as being from a non-White ethnic group.

### Qualitative themes

Three themes were identified; these focused on: the role of family members in encouraging screening attendance, the current phrasing around types of tobacco use and lung screening eligibility, and non-smokers. It was considered prior to analysis that the MPSG data may have indicated a preference for contact and invitational materials based on societal segmentation. However, the data indicated that there were no significant differences identified in the responses according to MPSG. This may be due to the small number of responses per MPSG category, and a study including more participants may show a different perspective. The themes identified in the data are discussed in more detail below.

### Role of family members

Family members play a variety of roles in terms of supporting their relative’s health^16^. Participants who smoked, reported that family members could probably influence them to attend lung screening, as in many cases they had also encouraged them to attend health screening in the past:

*‘I wouldn’t have gone to the doctors about my mental health issues… and she (FM) just went ahead and booked me an appointment.’* (PSP8)

Family members were often influential in encouraging attendance for health screening or helping to adopt healthy behaviors:

*‘He does keep me on track with my no smoking, he tells me off!’* (PSP13)

*‘I stopped smoking because I was proving a point to my dad*.*’* (PSP23)

Some family members said that their parent may attend lung screening if they went together. A family member’s ability to tailor their persuasive approach to their relative’s personality was considered to be a key factor in encouraging attendance. Family members stated that they knew how best to talk to their relatives. Some respondents reported that simply talking it through with family members would help them to see the benefits in attending lung screening:

*‘He would say, “it will give you peace of mind”, that’s the way he’d speak to me.’* (PSP3)

Lung screening eligible participants appeared more likely to engage if encouraged by a relative, with conversations regarding screening opportunities and support to attend being seen as a key first step. Greater information about screening and improved accessibility to that information was identified as useful, such as materials available in a wider range of languages and easier access to large print versions, which whilst identified as available, were not easily accessible.

### Smoking phraseology

When discussing invitational materials for lung screening, participants began to consider their smoking identities. Some of those who were currently vaping did not consider themselves a ‘smoker’, especially if they were using a vape as part of the quitting process. Whilst the criteria for screening included previous smokers, participants did not seem to identify with that aspect of the eligibility and focused instead on their current status. For example, some vapers, felt eligibility criteria that referred to ‘smokers’ or ‘previous smokers’, did not apply to them (even though many were previous smokers) as they no longer identified as a smoker and as such felt that this should be addressed:

*‘I think it should include vaping, it shouldn’t just say smoking.’* (FG1-1d)

It was also highlighted that other forms of tobacco use are often missing from the promotion of lung screening such as oral tobacco, which is rarely identified as a form of smoking behavior:

*‘As of now, I don’t vape or smoke, but I do use oral tobacco.’* (FGPPI)

### Lung screening eligibility

Lung screening eligibility was highlighted throughout the data collection by participants, and whilst this final theme was not a key focus of the study, the data suggest it highlights some important points for consideration in lung screening communications. Family members and people who smoke were concerned about current eligibility for lung screening. The restrictions to people who smoke were considered to be unfair, with many stating that people who do not smoke were also at risk of lung cancer^20^ and so screening should be open to all:

*‘There’s a lot of people get lung cancer who haven’t smoked a cigarette in their life isn’t there?… everybody gets it. Cancer doesn’t care.’* (PSP4)

There were also concerns raised about individuals that work in industries that affect lung health who may not be included in eligibility criteria (although potential industries were not directly identified). It was noted that many of the promotional materials for lung screening come from the Roy Castle Foundation^21^, but that as a non-smoker Roy Castle himself under the current criteria would not have been eligible for lung screening:

*‘He wasn’t a smoker himself, but he worked in smoking situations so I think maybe consideration should be given to people that maybe live with a smoker.’* (FG1-1)

Family members expressed concerns about years of exposure to passive smoking on their health and felt that this was not considered in current access to lung screening:

*‘They could have cancer or some smoking-related disease associated with the lungs … not necessarily brought on by themselves but by other people being around them.’* (FG1-2)

Age was considered by many to be a way to address eligibility issues, with potential screening participants being identified based on age as opposed to smoking status:

*‘There must be an age where it gets more prominent in people.’* (PSP24)

However, there was confusion about the cut off age of 75 years, and the rationale underpinning this:

*‘What’s the rationale between stopping at 75?…they’re giving up on me or if I haven’t got lung cancer by the time I’m 75 I’m never going to get it?’* (PSP24)

## DISCUSSION

Our findings show that numerous factors still need to be addressed to improve informed participation in a national lung screening program, with a key factor to be further explored being the role of family members and their influence and importance in terms of smoking behavior. A recent study^22^ exploring the key factors that prevent uptake of cancer screening described a range of issues that the family members in this study claim to support their relatives with. Increased dialogue with family members may help identify the best approaches to increasing attendance at lung screening.

Never smokers (individuals who have never smoked tobacco) are not eligible for lung screening and this was often highlighted by participants as a concern. Those who had smoked reported that it was unfair to not include never smokers in screening, whilst some family members who were concerned about passive smoking also raised it as an issue. The eligibility of never smokers in lung screening provision therefore needs to be clearly addressed and justified in participant literature to prevent it being a distraction. Despite the relatively low incidence in never smokers, individuals in this category can feature in the media. Our study identified this as a key concern for some previous passive smokers^23^. In addition, the justification for the chosen age range within the eligibility criteria needs to be much more explicitly outlined and justified in lung screening promotion.

E-cigarettes are often used as part of the quitting process^24^ and are identified by many as a solution to quitting. Once individuals begin vaping, some no longer considered themselves to be a ‘smoker’ and seem to not think of themselves as a previous smoker (with this part of the eligibility often being ignored). This is another area where information needs to be clear about the phrasing of smoking practices and how these can best reflect all forms of nicotine use.

### Limitations

Small qualitative studies such as this face challenges with regard to limited generalizability. As such the findings from this study are provided to offer a starting point for the discussion of potential invitational materials, not a definitive answer. Discussion in focus group settings may also be affected by response bias, in which members simply agree with each other to avoid confrontation. In addition to this, due to the positive social perception of health screening, response bias may have also been a factor in individual responses regarding lung screening, with participants wanting to be viewed in a positive light. As such there are limitations around the reliability of the results. However, the two-step process of the study data collection does allow for some respondent validation in an attempt to address these issues.

## CONCLUSIONS

Family members are a mostly untapped resource in health promotion and a more detailed study involving family members and lung screening promotion may be useful. Eligibility is focused on smoked tobacco, but this is clearly not understood by some potential participants, with confusion about what confers risk and therefore eligibility. Equally, it is important to explain why people may not be eligible, whether they are at low or very low risk, e.g. passive or never smokers. Our study has highlighted a number of significant issues that could be addressed in future research.

## Data Availability

The data supporting this research are available from the authors on reasonable request.
